# Application of a Brain-Inspired Spiking Neural Network Architecture to Odor Data Classification

**DOI:** 10.3390/s20102756

**Published:** 2020-05-12

**Authors:** Anup Vanarse, Josafath Israel Espinosa-Ramos, Adam Osseiran, Alexander Rassau, Nikola Kasabov

**Affiliations:** 1 School of Engineering, Edith Cowan University, Perth 6027, Australia; a.osseiran@ecu.edu.au (A.O.); a.rassau@ecu.edu.au (A.R.); 2 Knowledge Engineering and Discovery Research Institute, Auckland University of Technology, Auckland 1010, New Zealand; josafath.ramos@aut.ac.nz; 3 Intelligent Systems Research Centre, Ulster University, Magee Campus, Londonderry BT48 7JL, UK

**Keywords:** biomimetic pattern-recognition, neuromorphic olfaction, electronic nose systems, spiking neural networks (SNNs), SNN-based classification

## Abstract

Existing methods in neuromorphic olfaction mainly focus on implementing the data transformation based on the neurobiological architecture of the olfactory pathway. While the transformation is pivotal for the sparse spike-based representation of odor data, classification techniques based on the bio-computations of the higher brain areas, which process the spiking data for identification of odor, remain largely unexplored. This paper argues that brain-inspired spiking neural networks constitute a promising approach for the next generation of machine intelligence for odor data processing. Inspired by principles of brain information processing, here we propose the first spiking neural network method and associated deep machine learning system for classification of odor data. The paper demonstrates that the proposed approach has several advantages when compared to the current state-of-the-art methods. Based on results obtained using a benchmark dataset, the model achieved a high classification accuracy for a large number of odors and has the capacity for incremental learning on new data. The paper explores different spike encoding algorithms and finds that the most suitable for the task is the step-wise encoding function. Further directions in the brain-inspired study of odor machine classification include investigation of more biologically plausible algorithms for mapping, learning, and interpretation of odor data along with the realization of these algorithms on some highly parallel and low power consuming neuromorphic hardware devices for real-world applications.

## 1. Introduction

Biological sensory architectures found in nature exhibit remarkable computational abilities and have the capacity to perform efficiently and accurately, even under noisy conditions [1]. Pursuing the idea of replicating the same efficient style of computation, foundational research [2] by Persaud and Dodd aimed to develop an artificial olfactory system based on the functional blocks of the biological olfactory pathway. While this study introduced the notion of using a sensor array as the sensing front-end and established a general architecture for electronic nose (e-nose) systems, the implementation of conventional statistical methods to process multivariate time-series sensing data imposed limitations due to substantial computational latency, high power requirements, and poor classification performance and reliability [3].

The introduction of neuromorphic engineering brought a paradigm shift in the electronic sensing domain [4]. The low-power bio-inspired approach significantly reduced the data overhead by using a spike-based sparse representation of information, which could be processed much faster than traditional methods [5,6]. Promising results obtained by applying neuromorphic concepts for vision and auditory sensing stimulated research into neuromorphic olfaction. Furthermore, the development of bioinspired learning methods such as spike-timing-dependent plasticity (STDP) and advancements in the utilization of spiking neural networks (SNN) for classification of odors based on temporal spiking information, reinforced the applicability of this approach for the development of robust and real-time electronic nose systems [3,6].

Drawing inspiration from the neurobiological architecture of the olfactory pathway, Koickal et al. in [7] implemented the first adaptive neuromorphic olfaction chip that consisted of a chemosensor array front-end, a signal conditioning module, and an SNN for processing and classification. While this study mainly focused on achieving a high degree of bio-realism in emulating its biological counterparts, it did not quantify the classification performance of the model, and its application in a real-world scenario was not plausible due to several issues such as component mismatch inherent in analogue designs [3,8]. Following this research, a number of neuromorphic implementations, such as [9,10,11,12,13,14,15], emerged that focused on detailed modelling of their biological counterparts, but their practical application was limited due to factors such as complexity of the system (e.g., large sensing array from the NEUROCHEM project [16]), strict operating constraints, and limited classification performance [3,6].

Recent developments in neuromorphic olfaction have focused on leveraging the inherent advantages of the spike-based data representation to develop practical e-nose systems where key aspects such as data-to-spike encoding techniques, utilization of SNNs for pattern-recognition, and implementation of these models on low-power hardware are emphasized [17,18,19,20,21,22]. However, these neuromorphic models have mainly focused on data transformation based on biological spike encoding architectures, while overlooking the overall performance of the system to identify target odors with minimum computational resources and latency.

While the biological olfactory pathway plays a crucial role in the generation and transformation of odor information, biological studies have indicated that the bio-computations in higher-brain areas of the olfactory cortex have profound implications on how odors are classified [23,24,25]. Hence, through this investigation, we focus on utilizing the neuromorphic approach to develop a 3D SNN model for pattern recognition in an e-nose system. Contrary to other studies that mainly focus on emulating biological techniques for encoding real-valued sensor responses into spiking data [7,12,19,21,22], we base our approach on utilizing standard encoding methods and focus on implementing a brain-inspired SNN model for classification of spatiotemporal odor information. Given the fact that neuromorphic models enable rapid processing [4,6,26], the development of the SNN classifier will also focus on exploiting this inherent advantage to minimize the latency incurred during the classification task and for better understanding of the data. Another key aspect investigated in this study includes the classification of raw sensor responses without the requirement for pre-processing or feature extraction to overcome any processing and latency overheads resulting from these steps.

## 2. Methods and Materials

### 2.1. System Architecture

**Figure 1:**
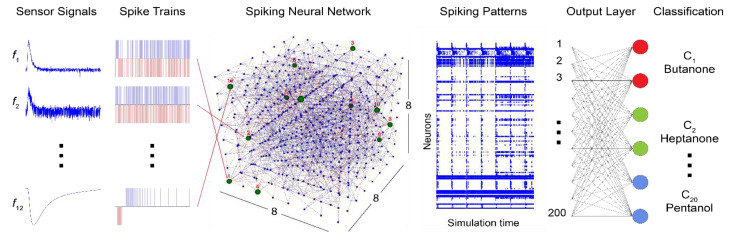
Structure of the proposed brain-inspired spiking neural network architecture for odor classification. The responses from the 12-sensor array are encoded into spiking data and presented to an 8 × 8 × 8 3D spiking neural networks reservoir (SNNr). The spiking patterns resulting from the computations within the 3D SNNr are used by the output layer consisting of 200 neurons for odor identification.

The bio-inspired classifier model proposed in this study is designed using the NeuCube framework [27,28], a 3D brain-inspired evolving connectionist system (ECOS) [29,30]. One of the key features of NeuCube, crucial for this implementation, is its unified platform comprising of a data-to-spike encoder, a spiking neural network reservoir (SNNr) for deep learning of input spike trains, and an output/classification module which can generate/evolve new output neurons to accommodate new input data or classes of data [31]. Taking inspiration from the biological olfactory pathway and based on the aforementioned NeuCube modules, our model is comprised of three key stages of electronic nose data processing: transformation, learning, and classification. Promising results have been obtained using NeuCube models for various applications [27,30,32], providing evidence of the robust classification capabilities of the SNN framework, even under potentially noisy and multidimensional spatiotemporal data. These results make NeuCube one of the ideal candidates to explore the applicability of the brain-inspired SNN model for the classification of raw sensor responses. A conceptual model diagram of the proposed system is shown in Figure 1.

This study utilizes the benchmark e-nose dataset [33] consisting of real-valued signals recorded at 2 Hz over 300 secs using a 12-sensor array exposed to 20 different chemical compounds. Since one of the objectives of this work is to overcome the requirement of pre-processing and feature extraction, the raw sensor responses along with relative resistance curves and exponential moving averages are used as an input to the data-to-spike encoder. Without any pre-conditioning, the optimal encoding of task-relevant information from the original sensor response curves, including steady-state and transient features, is pivotal to obtain reliable classification results. The sensor responses were encoded into spiking data using an encoder with built-in optimization of encoding parameters based on the error metrics between the original and reconstructed signals.

The spiking information is propagated through the 3D SNNr for deep learning and classification [27,28,30]. The SNN is initialized as a 3D reservoir, also called the “Cube”, with leaky integrate-and-fire (LIF) neurons connected in a recurrent structure following the principles of a small-world network. Learning within the SNN model is implemented in two phases: In the first phase, the input spike sequences are propagated through the network, and an unsupervised learning method, such as STDP, is implemented resulting in modifications of the neuronal connections based on the time that pre and postsynaptic neurons fire. Based on the neuron’s activation patterns, the SNN learns to identify similar odor stimuli. In the next stage, the dynamic evolving SNN (deSNN) [31] and supervised learning is implemented as the output classification module, where output neurons are trained to classify the input spiking data that activate spatio-temporal patterns in the SNN cube based on predefined labels for odor classification. deSNN has an evolving structure, which evolves (creates) new output neurons for new data and classes, added incrementally to the system.

Once the training stage is completed, the connection weights are retained as long term memory, and the trained model can be used as a back-end classifier for an electronic nose system, having in mind that such a system is adaptive to learn and classify new data in an incremental way by generating new output neurons in the deSNN classifier. The SNN model developed using the NeuCube framework can be deployed on a cloud or hardware platform, such as the SpiNNaker [27], which is one of the crucial aspects for the development of a standalone electronic nose system.

### 2.2. Sensing System and Dataset Description

The benchmark electronic nose dataset, extracted from the CSIRO Data Access Portal [33], was used as an input dataset for the training and testing of the proposed SNN classifier. The measurements in the dataset were performed under laboratory conditions using the FOX 3000 electronic nose (Alpha M.O.S., Toulouse, France). The e-nose system, originally equipped with a 12-sensor array, comprises of six standard doped tin dioxide (SnO_2_) and six chromium titanium oxide (CTO) sensors and tungsten oxide (WO_3_) sensors. However, during the experiments (detailed in [34]) the CTO and WO_3_ sensors were replaced with six novel CTO based sensor arrays [35] that include five zeolite-coated and one uncoated CTO sensor. The modified array implements an additional transformation layer comprising of acid (or sodium) forms of zeolites over the porous CTO sensing element that enables the size and shape of odor molecules interacting with the sensor to be limited through pore size control and selective permeability [36].

During the measurements, the two arrays were housed in different chambers due to their different physical properties. The 12-sensor array was exposed to 20 different chemical compounds taken from four chemical groups: aldehydes, alcohols, ketones, and esters with five chemicals per group. Overall, the dataset consists of 200 data samples with 10 replicates for each sample recorded for a total of 300 s at a frequency of 2 Hz. A delay of 240 s was imposed between the samples for a cleaning procedure where dry zero grade air was used to remove any residual odor sample from the sensor chambers and the sensors were allowed to return to baseline. Additional details regarding the sensing system, laboratory conditions, the concentration of odors, and the measurements are described in [34].

### 2.3. Data-to-Spike Encoding

A Java-based data encoding tool included within the NeuCube framework is used to encode the temporal odor information into spike trains. The spike encoding stage is critical for this application because the original sensor responses consist of both useful information and noise, and without any pre-processing or feature extraction, the encoding logic needs to be able to preserve the critical discriminative information along with a sparse representation of the sensor response curves. The effectiveness of the spike encoding method for classifiers, especially for olfactory systems, is generally evaluated based on a comparison between the original and the reconstructed signals using the error metrics and the overall SNN output, which in this case is the classification accuracy.

Among the different encoding schemes based on either rate or temporal coding, the encoder within the NeuCube framework uses temporal coding to represent the input information. The spike encoding algorithms integrated within the encoder are based on two different approaches:

1. Temporal contrast, where the temporal changes in the signal are encoded in the form of spike timing.
2. Stimulus estimation, a bio-inspired encoding method that generates unipolar spike trains to represent the original signal.

The temporal contrast-based encoding methods supported by the encoder include threshold-based representation (TBR), step-forward (SF) encoding, and moving-window (MW) encoding. Ben’s spiker algorithm (BSA) is the only stimulus estimation-based encoding method included in the NeuCube framework [27,30,37].

Based on the analysis and evaluation of different encoding methods presented in [37], SF encoding was chosen for this implementation because of its versatility and robustness. This approach is based on encoding the input signal within an interval around a moving baseline with a set threshold. Once the initial baseline is set to the initial signal value, a positive or a negative spike is generated when the subsequent signal value is either above or below the baseline and the threshold value. Along with the spiking output, the baseline is adjusted to the upper or lower limit of the threshold interval. Reconstruction of the signal from the spike-encoded data is derived by multiplying the encoding threshold by the summation of positive and negative spikes. The algorithmic approach for decoding is further explained in [37]. Features such as robust optimization and a straightforward decoding process make SF encoding an ideal candidate for this application.

Along with SF encoding, the SNN classifier was also tested for unipolar spike trains encoded using the BSA encoding method. The implementation of BSA encoding is abstracted from the response function of biological neurons and, hence, is the most biologically plausible encoding technique among those included within the NeuCube framework. This method utilizes a finite-impulse response (FIR) filter to encode analogue signals into spike trains. BSA encoding was developed with a primary aim of simplifying the decoding process, which is implemented by the convolution of spike trains with the filter coefficients. Based on the analysis provided in [37], BSA encoding may not be the ideal candidate for encoding rapidly changing signals, which is the case for electronic nose systems. However, we utilize this method in order to analyze the SNN-based classifier’s output for unipolar spike trains.

### 2.4. Learning and Odor Recognition in the Proposed SNN Architecture

The SNN architecture proposed in this research for deep learning and odor classification is based on the NeuCube framework. The NeuCube framework is a spatio-temporal data machine mainly developed to model and process spatio-and spectro-temporal brain data [27,28]. The framework principally consists of three main functional components: a data encoding module, a 3D SNNr module for deep learning, and an output/classification module.

The process of creating a NeuCube model for a given multivariable dataset takes the following steps:

1. Encode the multivariate input data into spike sequences: continuous value input information is encoded into trains of spikes.
2. Construct and train in an unsupervised mode a recurrent 3D SNNr, to learn the spike sequences that represent individual input patterns.
3. Construct and train in a supervised mode an evolving SNN classifier to learn to classify different dynamic patterns of the SNNr activities that represent different input patterns from the multivariate data that belongs to different classes.
4. Optimize the model through several iterations of steps (1)–(3) above for different parameter values until maximum accuracy is achieved.
5. Recall the model on new data.

### 2.5. Experimental Framework

In this research, we utilized the JNeuCube for the classification experiments and the NeuCubeFX for visualization and analysis of the results. Both tools are Java implementations of the NeuCube architecture developed at KEDRI (http://kedri.aut.ac.nz) and now available on the cloud (www.neucube.io).

Similar to other machine learning techniques, the accuracy of the NeuCube models depends on the correct selection of the parameters for the methods and algorithms implemented. A major issue with NeuCube models is the optimization of the numerous parameters, which could be over 20 depending on the methods and algorithms selected. Besides the optimization of the encoding process, we optimized seven of the most important parameters related to the neuron model, the unsupervised and supervised learning, and the classifier. Since testing for different values for all possible combinations was impractical, we implemented a differential evolution-based (DE) [38] optimization process. Table 1 describes the DE and NeuCube parameters used in the optimization process, along with their boundary values.

The objective function was maximizing the average classification accuracy of 10 NeuCube models produced with the same set of parameters. For each candidate solution (set of 7 NeuCube parameters), the algorithm created 10 NeuCube models with random connection weights (uniformly distributed in a range [−0.1, 0.1]) and random location (uniformly distributed in the range [1, number of neurons in the hidden layer]) for the inputs. For each model, the algorithm randomly splits 70% of the data for training and 30% for testing, ensuring the same number of samples for each class of the dataset are selected. In the subsequent stage, each model was assessed using K-fold cross-validation on the training set (training accuracy), later fitted using the training set again, and finally evaluated for generalization using the testing set (testing accuracy).

The quality (fitness) of a model was assessed by calculating the average of the training and testing accuracy. The aim, by using such an approach, is to produce SNN models that are unbiased while tuning their hyperparameters using a 5-fold cross-validation scheme on the dataset for training and models that have high generalization using the dataset for testing (unseen dataset). Using only the classification performance of the SNN model for the testing set would produce models with high predictive skills but poor generalization skills, or models that can only predict the dataset for testing (30% of the data). The step-by-step algorithmic implementation for validating each candidate solution is shown in Algorithm 1. The validation process ensures that each NeuCube model produced has similar accuracy using the same set of parameters.
**Algorithm 1** Differential Evolution1:data = read(data)2:#tune model hyperparameters3:parameters = … {parameters refer to a population of the DE}4:numNeuCubeModels = …5:k = …6:**for** params in parameters **do**7:      paramSkills = list()8:      **for** n in numNeuCubeModels **do**9:                    train, test = split(data)10:                    skills = list()11:                    **for** i in k **do**12:                       fold_train, fold_val = cross_validation_split(i, k, train)13:                       model = fit(fold_train, params)14:                       skill_estimate = evaluate(model, fold_val)15:                       skills.append(skill_estimate)16:                    **end for**17:                    skill_train = summarise(skills)18:                    model = fit(train)19:                    skill_test = evaluate(model, test)20:                    paramSkills.append((skill_train + skill_test)/2)21:      
**end for**
22:      paramSkill = average(paramSkills)23:**end for**

## 3. Results

### 3.1. Input Data Encoding and Optimization

**Figure 2:**
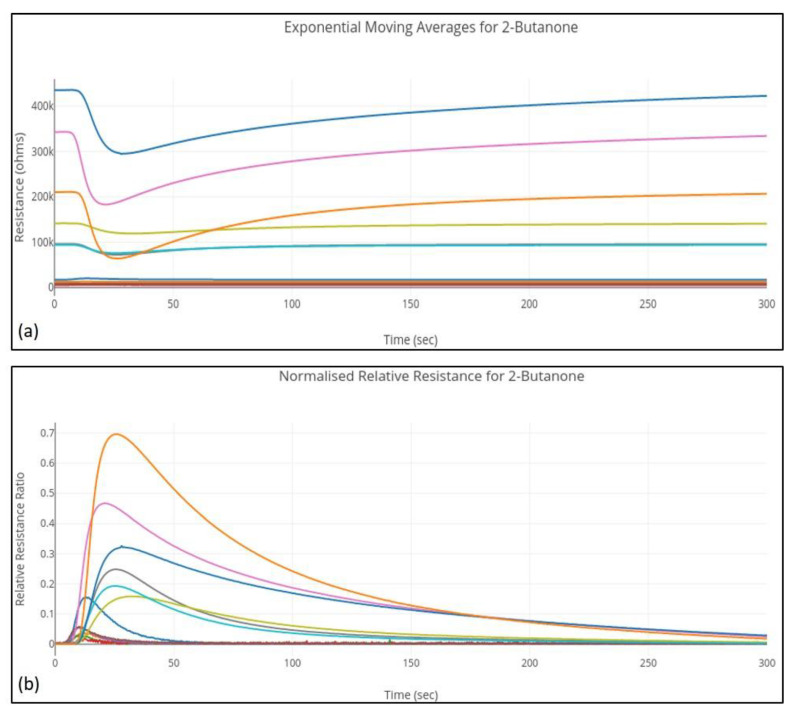
Feature sets for 2-Butanone sample. (**a**) Exponential moving averages. (**b**) Normalized relative resistance.

While, based on the objectives of this study, we primarily used raw sensor responses as input to the spike encoder, we also utilized feature-extracted data for spike encoding to compare the overall performance of the SNN model. In this case, we used the two most commonly used features mentioned in the e-nose literature:

- Normalized relative resistance features based on the following mathematical model:
  $$R_{norm}\left(x\right)=\frac{R_{i}-R_{0}}{R_{max}-R_{0}}$$
  where, $R_{norm}\left(x\right)$ is the normalized relative resistance for sensor x, $R_{i}$ is the measured resistance of sensor x at instance i, and $R_{0}$ and $R_{max}$ are the baseline and maximum resistances.
- Exponential moving average, a smoothing technique based on the mathematical model defined in [39] and a smoothing factor α = 0.5 selected based on the sampling frequency and its implementation shown in [34]. An example of the feature-extracted curves for a 2-Butanone sample is shown in Figure 2.

These signals are provided as input to the Java-based data-encoding module within the NeuCube framework. As discussed in Section 2.3, the continuous time-series input signals are encoded into spike trains using the SF and BSA algorithms. A detailed description of the algorithmic implementation of these encoding techniques is provided in [37]. The SF encoding utilizes a fixed parameter, the threshold value, along with a moving baseline to generate positive or negative spikes. The implementation of the BSA algorithm uses a much more complex logic using finite impulse filters resulting in three key parameters, the order of the filter, its cut-off value, and the threshold, to generate a unipolar spiking output. In order to retain task-relevant information from the original sensor responses, it is vital to use the optimum values of these parameters while encoding the sensor response signals into spike trains.

The efficacy of data-to-spike encoding is determined by reconstructing the signal using decoding algorithms corresponding to the encoding technique and comparing the recovered responses with the original signal. An optimization process is implemented to determine the best-fit values of encoding parameters that maximize the accuracy of signal recovery. This is established by calculating the error metrics between the original and the reconstructed signals.

**Figure 3:**
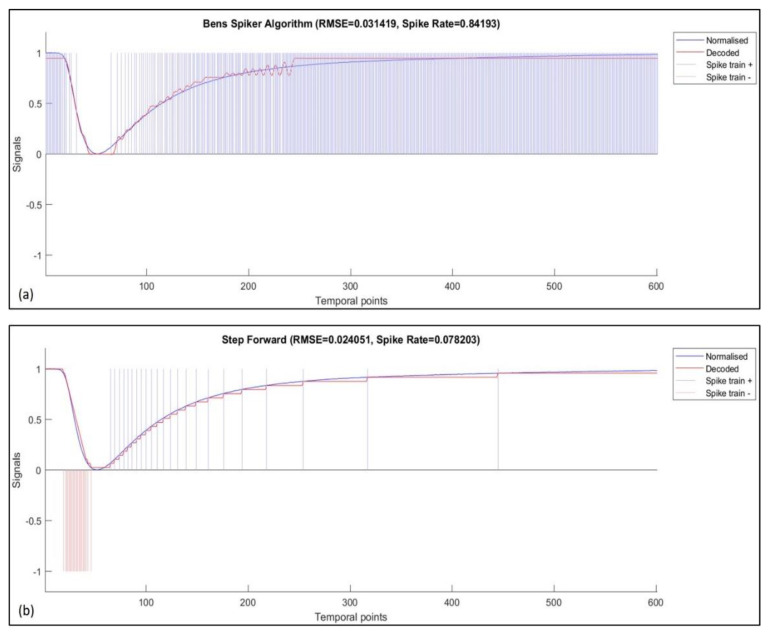
Spike-encoded data for sensor ten responses when exposed to 2-Butanone. (**a**) BSA encoding, (**b**) SF encoding.

Among the various candidate error metrics, this implementation uses root-mean-square error (RMSE) for parameter optimization. RMSE is defined in [37] as

$$\mathrm{RMSE}=\sqrt{\frac{\sum_{t=1}^{N}{\left(r_{t}-s_{t}\right)}^{2}}{N}}$$

where a summation of modelling errors between the original signal, s, and reconstructed signal, r, for a total of N time points is calculated and minimized. For SF encoding, an optimum value of the threshold parameter is determined using a grid search approach. As the BSA technique depends on multiple encoding parameters, a differential evolution (DE) process is implemented for parameter optimization. The optimization process is applied on each sensor channel in the 12-element array response and for each odor sample. Figure 3 illustrates the spike-encoded data using both SF and BSA algorithms for sensor 10 when exposed to a 2-Butanone odor sample.

### 3.2. NeuCube Model Optimization

The DE approach was found to be the most efficient (in terms of the number of iterations and accuracy of the solutions) optimization tool for finding the best NeuCube parameters. The models implemented using these optimum parameters enable the classification of 20 chemical compounds with an overall accuracy greater than 90%. The analysis presented in this section is based on the highest overall accuracy result, which was obtained for the classification of the 20-class dataset while operating on the SF encoded original sensor responses.

**Figure 4:**
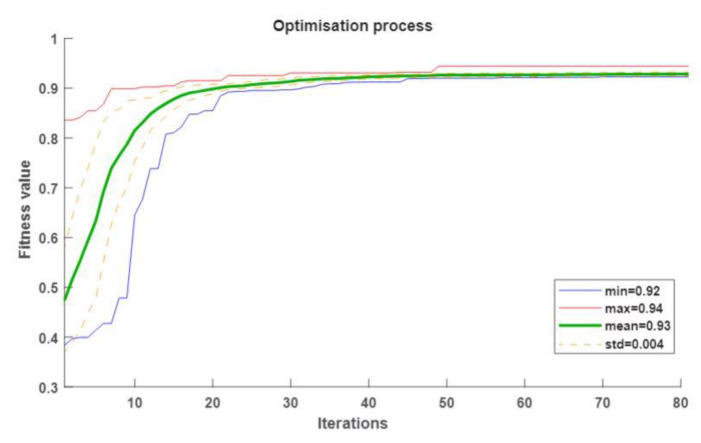
The optimization process of the parameters of the SNN model showing the accuracy obtained over 80 iterations.

The optimization process rapidly started producing agents with an overall accuracy greater than 90% after the 10th iteration. However, the whole population reached that percentage after the 31st iteration with a standard deviation of ± 0.6%, which is a good indicator of the stability of the optimization process. The algorithm found the best solution (set of parameters) with a 94% overall accuracy in the 49th iteration; after that, the population’s overall accuracy average improved very little from 93% to 94%. Figure 4 shows the overall accuracy metrics at each iteration of the DE, and the summary of the parameters in the last iteration is listed in Table 2.

We can observe that the DE approach generated candidate solutions in which parameter A− of the STDP was eight times higher than the parameter A+, thus producing SNNs that could exhibit inhibitory behavior, i.e., more negative than positive weights. In the best solution, we can observe that it showed a negative value for the parameter A+. Although negative values of A+ or A− have no biological meaning in the STDP; in this particular case, a negative A+ and the higher value of A− regulated the firing activity preventing saturation and lack of temporal patterns.

### 3.3. SNN Modelling

Similar to any commonly used artificial neural network (ANN) architecture, the NeuCube model is arranged in layers. However, some specific properties of the NeuCube suit the processing of spatial and temporal data. In this research, the set of 12 sensor signals encoded into spike trains (predictor temporal variables) is presented to the input layer. The selection of the input neurons can be either done using a brain template, such as for electroencephalogram (EEG), functional magnetic resonance imaging (fMRI), and other data [27,28,30], or can be done by a preliminary analysis of the dynamics of the input variables so that variables with similar dynamics can be located closer in the 3D SNN architecture [40]. Each neuron in the defined input layer of neurons distributes a spike train to the neurons in the middle layer of recurrently connected neurons.

The middle layer is a set of 8 × 8 × 8 leaky integrate-and-fire neuron models (3D SNN) [41] that capture deep spatio-temporal relationships among the temporal variables. The connections among the neurons follow the principle of small-world networks [42], forming recurrent connections that process streams of data and learn temporal patterns as a result of the network’s firing activity. In the NeuCube architecture, every neuron in the middle layer is also connected to a neuron in the output layer. Every output neuron and its connections coming from the middle layer represent the spatio-temporal activity in the SNNr corresponding to a single sample. In this particular case, 200 neurons, corresponding to the number of samples in the dataset, formed the output layer. Merging of output neurons based on their connection similarity can be applied so that a single output neuron can represent not just a single sample but a whole cluster of similar (in space and time) samples [30,31].

### 3.4. Deep, Unsupervised Learning in the NeuCube Model

After the optimization process, we generated a new SNN model applying the parameters of the best solution. The NeuCube has two features for analysis, the firing activity and the recurrent connection neurons, which describe temporal and spatial patterns, respectively. In this section, we analyze both features before and after the unsupervised training.

**Figure 5:**
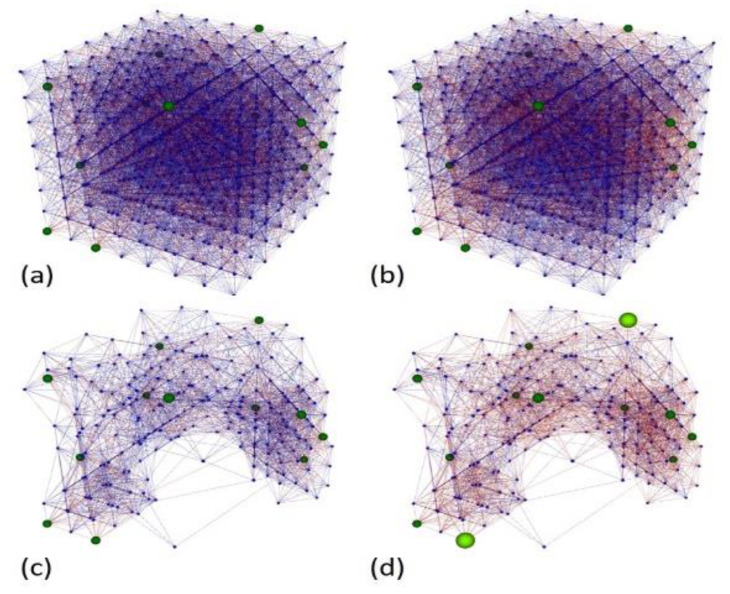
NeuCube model (**a**) before and (**b**) after training. Functional neurons and connections (**c**) before and (**d**) after training. Green dots indicate the input nodes, and brighter green dots indicate that the node fired a spike at the particular time of the snapshot. Blue and red lines indicate positive and negative connections, respectively. Each input odor sample is learned as a deep spatio-temporal pattern of connections.

Additionally, we implemented a novel pruning method that removed neurons and their connections and did not emit any spike while feeding the SNN with the whole dataset. Removing useless elements improved the SNN performance in terms of processing time and memory and allowed better visualization of the information trajectories formed during unsupervised learning. Samples belonging to the same class shared similar trajectories that were different enough from those formed with samples belonging to other classes for classification. Figure 5 shows the complete and pruned best NeuCube model before and after training.

After the unsupervised training, we observed an expected inhibitory behavior of the SNN because the value of A+ was lower than A− even though it was negative. Indeed, on average, the DE produced lower values for A+ than A−. Inhibition reduced the firing activity of the reservoir neurons. Before training, the 97,902 spikes coming from the input data (200 samples) produced 216,397 spikes (average firing rate = 0.0035), and after training, the same data produced 207,439 spikes (average firing rate = 0.0034).

The firing activity is relevant to the supervised training because it forms the values of the connections between the reservoir and the output layer. After feeding the SNN and applying the deSNN, the KNN uses those weights for classification. We can assume that most of the weights reached negative values due to the low firing rate and a higher value of the Drift− parameter.

**Figure 6:**
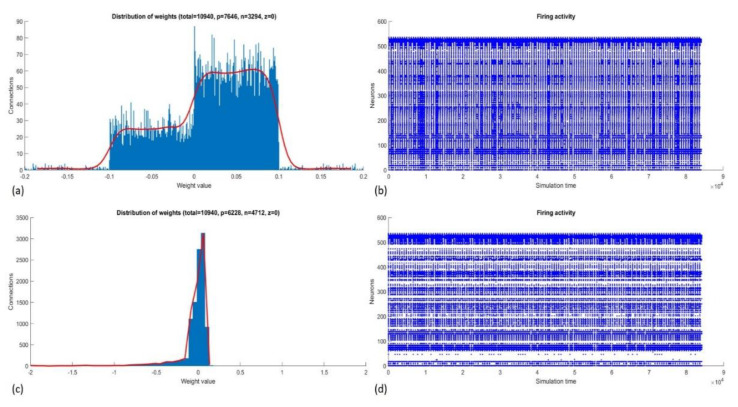
NeuCube weights (**a**) and firing activity (**b**) before training. Subsections (**c**) and (**d**) show changes in the weights and firing activity after training.

Inactive neurons in the SNNr, which did not have active connections with other neurons, were suspended from further use (temporarily pruned), which reduced the size of the reservoir from 512 to 223 neurons and the number of connections from 10,940 to 2881. This accelerated the processing time and reduced the memory use, especially during the supervised training and classification stages. As mentioned in Section 3.3, all neurons in the middle layer (reservoir) were connected to every neuron (sample) in the output layer. Therefore, the pruned SNN formed 223 instead of 512 output connections per sample, a significant reduction of the dimensionality of the space for classification. Figure 6 shows the number of positive and negative connections and their distributions before and after training. The number of connections that changed after training is listed in Table 3.

### 3.5. Classification Performance and Analysis

Once the optimized parameters were calculated, the SNN-based classifier was tested using a balanced 5-fold cross-validation strategy for both cases: the 20-class dataset (identification of individual odors) and 4-class dataset (classification based on chemical groups), which enables the model to test for generalization and to determine its performance for larger datasets with limited class labels. For each scenario, a total of 140 observations were used for training, and the model was tested based on the remaining 60 samples. The overall latency observed for training the NeuCube model, including supervised and unsupervised training, was between 3.5 and 4 s. The classification performance of the SNN model for each scenario is listed in Table 4.

In general, the highest overall accuracy was achieved using SF encoding and for the classification of the 20-class dataset. The classifier was able to identify 20 individual odors based on the original sensor responses encoded using the SF algorithm with 94.5% accuracy and the highest candidate accuracy of 96% during the 5-fold cross-validation. Under similar conditions, the classification rate for feature sets, including exponential moving averages and normalized relative resistance, was 93% and 87.5%, respectively. The classification results for the 20-class dataset using BSA encoding were mostly in the range 77% to 80%, with the best candidate solution of 83% for the exponential moving averages feature set. Misclassifications were typically observed for odors that belong to the same chemical group involving overlapping or closely positioned features (e.g., acetone and 2-heptanone). A maximum processing latency of 950 ms was recorded for a trained NeuCube model to provide an identification result.

Considering these results, we can infer that:

- The classification results obtained using SF encoding were significantly higher than the results obtained using BSA encoding. The bipolar spike trains generated by SF encoding represented the signal changes more accurately and enabled the implementation of inhibition within the network. Moreover, lower AFR and RMSE resulting from SF encoding ensured that any existing noise was suppressed and saturation of the SNN due to excess spikes was avoided. Comparatively, BSA encoding resulted in higher RMSE and average firing rate (AFR), thus resulting in errors for rapidly changing and plateau characteristics in sensor signals. Additionally, BSA encoding generates unipolar spike trains, hence, restricting the use of negative connectivity weights (inhibition).
- One of the aims of this study, to implement classification on the raw sensor responses without any pre-processing or feature extraction, was achieved. The SNN model, in fact, obtained the highest classification result of 94.5% for the original sensor data in comparison to other feature sets. These results indicate that the pattern-recognition performance of the SNN model is robust to noise. While feature extraction is useful in representing signal characteristics such as the maximum and relative resistance values, they often represent piecemeal information of the entire dynamic process that can be crucial for bioinspired classification approaches like the one presented in this study.

Secondary experiments were based on the 4-class dataset for the identification of the chemical group of compounds. In this case, a maximum classification accuracy of 84% was achieved using the SF encoding for the exponential moving averages feature set. Classification accuracy for other feature sets using SF encoding was recorded as between 74% and 80%, whereas feature sets encoded using BSA achieved accuracies between 60% and 68%. One of the main reasons for the lower classification rates is the lack of differentiating features between the classes. For example, the response characteristics of sensor 1 to 6 among all four classes were almost similar. These results indicate that the SNN-based classifier is functional, but the classification of the 4-class dataset is a non-trivial problem and would require an alternate strategy or enhanced dataset to achieve highly accurate results.

Although studies based on traditional machine learning methods for odor classification of the same dataset have claimed to have achieved high accuracy [19,34], these methods impose substantial computational and power requirements. Moreover, these techniques often require complex processing constructs and iterative training, resulting in considerable latency to provide recognition results, and the generalization capacity may also be limited [3,18]. Other neuromorphic approaches based on the same datasets have either focused on implementing data transformation based on the biological olfactory pathway or hardware-friendly application. Hence, these approaches differ vastly in terms of encoding techniques and application of SNN for classification, thus making a direct comparison impractical. However, when evaluated against the spike-based approach described in [17,18,19], the 3D SNN model produces comparable results even when applied on original sensor responses without using any pre-processing or feature extraction. Moreover, a trained NeuCube model was able to provide a recognition result within a maximum processing latency of 950 ms on a medium specification desktop computer, inclusive of latencies resulting from software-based input/output and other programming constructs. A hardware implementation would almost certainly result in a reduction in latency to the sub 100 ms range, enabling true real-time response.

## 4. Conclusions and Discussion

In this study, we present a neuromorphic classifier based on brain-like information processing principles for implementation in electronic nose systems. This research investigates two critical aspects of olfactory data classification: (1) implementation of an SNN model based on the computing principles in higher brain areas responsible for the identification of odors and (2) utilizing the raw sensor responses for classification without any pre-processing or feature extraction. We demonstrate the classification capabilities of a 3D-SNN model implemented using the Java-based NeuCube framework by achieving an overall accuracy of 94.5% for the identification of 20 different odor compounds from the benchmark FOX e-nose dataset.

Feature sets, including the original sensor responses, exponential moving averages, and the relative resistance curves were encoded into spike trains using the data-to-spike encoding module within the NeuCube framework. The SF and BSA encoding techniques were used and parameter optimization based on minimizing the RMSE error metric was implemented to ensure that the discriminatory odor information was preserved. A differential evolution-based optimization was also implemented to obtain optimal NeuCube model parameters that can provide stable and maximum classification accuracy with minimum neural resources (number of neurons).

The classification performance of the SNN model was analyzed under different scenarios, including 20-class and 4-class datasets, spiking data encoded using either SF or BSA, and three feature sets. In general, the results obtained through this study indicate that the SNN model produced better results for SF encoded spiking data while operating on the original sensor responses. Along with encoding and neural network parameters, factors such as inhibitory behavior of the neural network and exposure to the entire dynamic process of the sensor responses have a direct impact on the pattern recognition capabilities of the model. The classification performance of the SNN model, when applied to the 4-class dataset, was limited to a maximum of 84%. In this case, the bioinspired classification logic could benefit from dimensionality reduction and other feature extraction strategies to further improve its performance.

An important feature of the proposed approach is that the developed system is evolving and can be further trained incrementally on new data, including new classes, without using old data. It can also be used to apply transfer learning, where a system trained on one set of odor data can be further trained on a new set of odor data that contains new information. A further study could explore these characteristics of the proposed approach along with exploring different mappings of the input data into the 3D SNN architecture for a better interpretation of the model and a better understanding of the spatio-temporal patterns captured in the data with reference to human odor perception.

The spiking models developed using the NeuCube framework can be deployed on SpiNNaker [32,43], a neuromorphic hardware platform. Future research based on this study can take advantage of the hardware compatibility to further reduce the processing latency, and hence, a real-time low-power classification back-end for an artificial olfactory system can be envisaged. Moreover, the SNN model can be also be deployed on the cloud-based platform for applications related to distributed e-nose sensing systems. Implementation of the SNN-based classifier for a real-world application and studying the model when deployed on a neuromorphic hardware platform are charted for future research.

## Figures and Tables

**Table 1:** Differential evolution (DE) and NeuCube parameters involved in the optimization process.

Method	Parameter	Description	Limits
DE	Population size	Number of candidate solutions (agents), usually 10 times the dimension of the agents	70
	Max generations	Maximum number of generations	100
	Crossover probability	A rate that increases the diversity of the agents	0.7
	Weighting factor	The differential weight between two agents to a third agent	0.1
LIF Neuron	Threshold	Threshold voltage value to emit a spike	0.01–0.5
	Refractory time	The time period during which a neuron rests after firing	2–10
STDP	A+	Determines positive synaptic modifications	0.001–0.05
	A−	Determines negative synaptic modifications	0.001–0.05
deSNN	Drift+	Determines positive synaptic modifications	0.001–0.05
	Drift−	Determines negative synaptic modifications	0.001–0.05
K-Nearest Neighbor (KNN)	k	The number of nearest neighbors	3–10

**Table 2:** The values of the seven SNN parameters and their corresponding classification performance obtained as a result of the DE optimization process.

		LIF	STDP		deSNN		KNN	
	Threshold	Refractory Time	A+	A−	Drift+	Drift−	K	Accuracy
best	0.03614	6	−0.00072	0.00369	−0.00051	0.01543	1	0.94
min	0.02836	3	−0.00076	0.00313	−0.00103	0.01056	1	0.92
max	0.03799	7	0.00123	0.00554	0.00964	0.03389	1	0.94
average	0.03248	5	0.00054	0.00442	0.00470	0.01764	1	0.93
std	0.00274	0.95	0.00049	0.00070	0.00255	0.00628	0	0.00

**Table 3:** Weights before and after training.

Model	Total	Training	Positive	Negative
Complete	10,940	Before After	7646 6228	3294 4712
Pruned	2881	Before After	1960 747	921 2134

**Table 4:** Classification performance of the 3D SNN classifier.

No. of Classes.	Feature Set	Accuracy (SF Encoding)	Accuracy (BSA Encoding)
20	Original Signals	94.5%	79%
	Exponential Moving Averages	93%	80%
	Normalized Relative Resistance	87.5%	77%
4	Original Signals	80%	66%
	Exponential Moving Averages	84%	68%
	Normalized Relative Resistance	74%	60%
